# Experiencing Socioeconomic Deprivation as a Carer in the United Kingdom: A Qualitative Study

**DOI:** 10.1111/hex.70502

**Published:** 2025-11-19

**Authors:** Megan Armstrong, Alma Jeri‐Wahrhaftig, Abi Woodward, Danielle Nimmons, Carolyn A. Chew‐Graham, Joanne Protheroe, Fiona Stevenson, Nathan Davies, Kate Walters

**Affiliations:** ^1^ Research Department of Primary Care and Population Health University College London London UK; ^2^ Wolfson Institute of Population Health Queen Mary University of London London UK; ^3^ School of Medicine Keele University Staffordshire UK

**Keywords:** carers, general practice, qualitative, socioeconomic deprivation

## Abstract

**Background:**

Informal carers compose approximately 7% of the UK population and, through their unpaid care, they make important contributions to society and the health care industry. Being an informal carer is higher in people experiencing socioeconomic deprivation; however, no qualitative research has explored the impact of this on the ability to provide care for those with long‐term conditions.

**Aim:**

To explore the experiences and challenges of being a carer whilst experiencing socioeconomic deprivation.

**Methods:**

Semi‐structured one‐to‐one interviews with adults experiencing socioeconomic deprivation (*n* = 12) living in London and Sheffield, United Kingdom. Participants were recruited through social media and community channels. Data were managed in NVivo and analysed using reflexive thematic analysis.

**Results:**

Three analytical themes were developed: (1) Economic insecurity including insecure housing and challenges with financial welfare leading to sacrificing the necessities such as healthy food, water and heating; (2) social and structural barriers such as a lack of opportunities for social mobility due to care impacting employment and educational attainment, as well as area‐based barriers and feeling unheard by professionals; (3) the emotional challenges and rewards of being a carer such as managing people with poor mental well‐being exacerbated by their socioeconomic situation, whilst finding their caring role meaningful.

**Conclusion:**

Carers experiencing socioeconomic deprivation face additional challenges and barriers in their ability to provide care such as more emotional work, making sacrifices of necessities due to financial constraints and feeling unheard. Policy changes are needed to better support this population financially and to enable social mobility, as well as development of interventions and support resources for carers to use to feel empowered and to maintain good well‐being.

## Patient and Public Involvement (PPI)

Our study included two PPI members who are experiencing socioeconomic deprivation; one who is a carer and one who has several long‐term health conditions. We recruited our PPI members to the team through advertisement in deprived areas. Both PPI members contributed to all stages of the study, including formulating the funding application and research questions, being a recruitment panel member of the researcher who collected the data, providing feedback and edits on topic guides and recruitment materials, and interpreting the data and generating themes. PPI members were paid following National Institute for Health and Care Research guidelines [1].

## 1 Introduction

As the population ages, people will live longer with long‐term conditions (LTCs) and the number of people living with LTCs and requiring care and support will also increase [2, 3]. In the United Kingdom, it is expected that informal or unpaid carers will be the primary support to people with LTCs [4]. Carers are typically close relatives of those they care for and provide emotional support, illness‐specific care and help with daily activities [5]. Carers are currently estimated to compose approximately 7% (4.9 million) of the UK population and their unpaid work save the UK government £162 billion each year [6].

Carers face difficulties caring for those with LTCs that impact their own health and well‐being such as anxiety, depression and emotional stress [7]. Reasons for this include witnessing the decline and suffering of those they care for, the removal of personal control, feelings of guilt and less opportunity for self‐care [8]. Carers are more likely to delay their own personal care and are less likely to access important social and emotional support due to caring responsibilities, jeopardising their well‐being [9]. Additionally, carers frequently report feelings of being ignored and underappreciated by health care providers, and experience long waiting times for services [8, 10].

Carers do not receive substantial financial compensation for their services [6, 10]. As of 2025 in the United Kingdom, carers can apply for ‘Carer's Allowance’ of £83.30 a week if they care for one adult for at least 35 hours a week, which is a maximum rate of £2.38 per hour [11]. Due to strict eligibility criteria including providing proof of at least 35 h of care; not working or earning over £196 per week; not in receipt of a pension; the person they are caring for must receive specific welfare and not be in hospital or residential care for over 28 days; and some individuals not classing themselves as carers or underestimating the care they provide, less than a third of UK carers claim for carer's allowance [12]. The lack of financial support for carers is particularly concerning given around 25% of carers live in poverty compared to 18% of non‐carers [13]. Poverty is defined as a household receiving 60% below the median income that year. Experiencing poverty is particularly higher in carers who care for more than one person, are aged between 25 and 44, are women or are from an ethnic minority background [13].

Socioeconomic deprivation is also associated with a higher incidence of informal caring potentially increasing economic insecurity and widening socioeconomic inequalities [14]. Socioeconomic deprivation refers to a lack of economic resources such as living in poverty, and also includes social factors such as education, occupation and the areas people live in [15]. There is likely to be added complexity caring for people experiencing socioeconomic deprivation. For example, people experiencing socioeconomic deprivation are five times more likely to have a psychiatric disorder, misuse substances, or self‐harm compared to the general population; these are associated with the later development of physical conditions such as renal disease, cancer and dementia [16]. People experiencing socioeconomic deprivation have highlighted the importance of their carers and those without a strong support system often find it harder to self‐manage their LTCs [17, 18]. A recent review on LTCs, caring and socioeconomic deprivation reported the focus of research tends to be on income as opposed to socioeconomic deprivation, and highlighted more evidence is needed on this topic particularly for informal carers [19]. To our knowledge this will be the first qualitative study exploring carers’ experiences of socioeconomic deprivation.

### 1.1 Aim

To explore the experiences and challenges of being a carer whilst experiencing socioeconomic deprivation.

## 2 Methodology

### 2.1 Design

This study used a qualitative design and semi‐structured interviews to gather, interpret and analyse data. This paper is reported according to the Consolidated Criteria for Reporting Qualitative Studies [20].

### 2.2 Recruitment

Participants were recruited between April 2022 and April 2023 from areas of deprivation (index of multiple deprivation of two or below [21]) in Sheffield and London. We recruited from these locations as they experience high levels of deprivation, are diverse in terms of ethnicity and cover the South and the North of the United Kingdom. Due to the area of deprivation being insufficient alone to determine individual characteristics of socioeconomic deprivation, and formulated with our PPI members, potential participants were also asked to ‘self‐identify’ with one of the following statements:

1. I have had no further education or training beyond the age of 16.
2. I feel like I am just about getting by or have difficulties affording the necessities I need in daily life such as housing, heating, food, clothing or access to the internet.

We recruited participants through several avenues including voluntary and community sector organisations (e.g., Age UK, Independent Age and Sheffield Mind), Housing Associations, carers networks and newsletters, social media platforms, and snowballing. Purposive sampling was used to ensure a diversity of participants in terms of gender, age and ethnicity. Criteria for eligible carers included caring for an adult (i.e., aged 18 years or above) who was diagnosed with a LTC. We focused on caring for adults within the context of age‐related health decline and the accumulation of LTCs that increases with age. All participants were offered a £50 voucher for their time.

### 2.3 Data Collection

Twelve semi‐structured interviews were conducted by an author, who is an experienced qualitative researcher of people experiencing socioeconomic deprivation (A.W.). Individual interviews were conducted one‐to‐one, in‐person at their home or virtually depending on the participant's preference. Participants self‐reported their age, gender, ethnicity, relationship to the person they were caring for and their conditions, level of education, housing status and annual household income. The interviewer did not disclose personal information about themselves but outlined the purpose of the study. The interviews lasted between 60 and 90 min and were guided by a topic guide covering key topics such as navigating access to support and health care and financial barriers to caring (Supporting Information 1). The topic guide was developed in consultation with our PPI members and was iteratively refined during the data collection. During the interviews, the participants were offered breaks as needed and all were followed up via email or phone to check on their well‐being given the sensitive nature of the topic. Interviews were audio recorded and later transcribed.

### 2.4 Data Analysis

We used Braun and Clarke's thematic analysis approach using their reflexive thematic analysis methods [22, 23]. Inductive coding and development of themes was conducted in NVivo by three authors (M.A., A.W. and A.J.‐W.) in an open and organic process that used trends and patterns in participant stories and experiences to create initial coding. The descriptive themes were then reviewed and refined by the whole team to develop analytical themes. While we do not view data saturation as a necessary criterion for qualitative research, we noted that no new themes were emerging in the later interviews, suggesting that we had achieved sufficient depth and breadth to address our research aims.

The expertise of the research team was multidisciplinary and included backgrounds in psychology, sociology and health inequalities as well as practicing GPs. M.A., the lead author, was a child‐carer and experienced deprivation, and D.N. provided insights from her own ethnic background, which enhanced data analysis. M.A. and A.W. have published reflections of conducting qualitative research with people experiencing socioeconomic deprivation [24].

## 3 Results

### 3.1 Participant Characteristics

Participants were mainly female (*n* = 10/12) and with an age range of 19 to 72 years old. Participants were Asian or Asian British (*n* = 5), White British (*n* = 4), Irish (*n* = 1), Black British (*n* = 1) and Turkish (*n* = 1). Five participants were based in Sheffield and seven were from London. Participants cared for their spouses (*n* = 3), parents (*n* = 6), adult children (*n* = 2) and both spouse and parents (*n* = 1). There were a range of health conditions of the person being cared for including stroke, heart disease, chronic pain, dementia, Parkinson's, diabetes, depression, anxiety and borderline personality disorder with on average four LTCs.

Five participants worked part‐time jobs in addition to caring, one participant was a university student, and six participants had no paid employment. The estimated annual household income was less than £15,000 for five participants, between £15,000 and £19,999 for two participants, between £20,000 and £29,999 for three participants, and between £30,000 and £39,999 for two participants with a range of two‐six people living in the households. Three participants owned their own home and nine were renting privately or from a Housing Association. Five participants had no further education beyond school, six had undergraduate degrees and one person had a master's degree.

### 3.2 Themes

**Figure 1:**
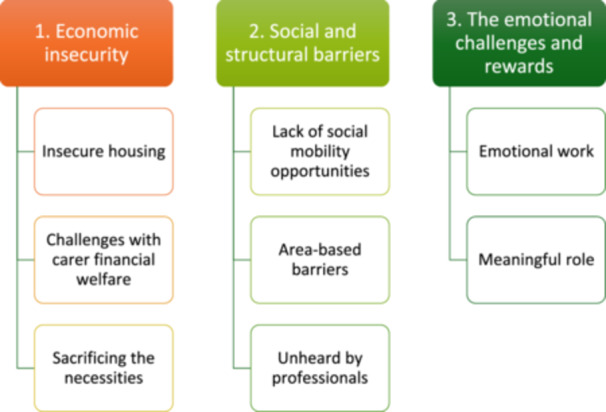
Developed themes exploring experiences and challenges of being a carer whilst experiencing socioeconomic deprivation.

Three main themes are developed, see Figure 1.

### 3.3 Economic Insecurity

#### 3.3.1 Insecure Housing

Carers in this study described how socioeconomic deprivation affected both themselves and the people they were caring for, often in interconnected ways. Anxiety around financial and housing instability was a common theme, with carers reporting stress both from managing their own resources and from concern about the living conditions and financial security of the person they cared for:

‘But he does get anxious about money and anxious about the heating. He got a letter today to say his direct debit going up. So, yes, he is anxious and I'm anxious about it too, I'm anxious about his housing, what happens if his tenancy runs out and it's not going to be renewed’.

[Female, Black British, Caring for ex‐spouse]

For carers who did not live the person they were caring for, worries about losing their own home were compounded by concerns about how this would impact the person they were caring for:

‘I'm always worried about my security. I'm no good to my dad if I've got no home. How can I help my father if I've got nowhere to live, and that's what it boils down to’.

[Female, White British, Caring for father]

There was little stability with some carers having to relocate to new areas or stay in houses that were unsuitable (e.g., due to size or condition). Unsuitable and unstable housing is particularly detrimental to carers who rely on the local community for support:

‘And we moved from house to house; I think we moved about nine times even…And because we don't have any choice…. we either had a nice house but in a bad area or we've had a good area but not a good – so like a damp house, or we had problems with the house, and it wasn't fit for us as a family of six’.

[Female, Asian, Caring for both parents and husband]

### 3.4 Challenges With Carer Financial Welfare

Financial assistance provided through the UK welfare system was described as challenging to access among many participants. Many carers worked part‐time or were unemployed, usually due to having to reduce their working hours to support their family, but despite being on a low income, were not eligible for carer's allowance:

‘I apply for the Carer's Allowance…If I've got less money then I am eligible for Carer's Allowance but my income doesn't match with them to get a Carer's Allowance…

[Male, Asian, Caring for wife]

Carers emphasised that navigating government support systems were a source of frustration, which required careful consideration of whether to work additional hours even when they were experiencing financial difficulties; this affected both their financial security and their capacity to provide care:

‘…at the minute because just the cost of living and obviously, the way the whole system is set up with getting benefits, I can only earn so much per week. So even in this cost‐of‐living crisis, I can't work extra to help me find the extra money so we have to cut back on things’.

[Female, White British, Caring for husband]

Another participant described avoiding applying for carer's allowance and accessing other financial support highlighting a lack of knowledge around what financial support there is:

‘Actually to claim for Carer's Allowance, it's so ridiculous you just don't want to. And so I've got this thing called Carers Credit and what that does is the government will pay your National Insurance contributions for you. But I didn't realise that until about ten years in really and then I had to sort of mention it…’

[Female, British Asian, Caring for both parents]

### 3.5 Sacrificing the Necessities

The impact of carers’ financial situation was a consistent concern for all. Occasionally the carers reflected on how the cost‐of‐living crisis had exacerbated their situation. Whilst providing unpaid care will impact many carers’ financial situation, this population do not have access to financial support from savings or wider family and so often had to cut back on necessities, which impacted them and, by extension, the person they cared for. The participant below describes how this has impacted their diet:

‘With the inflation at the moment…I haven't really been able to make anything nutritious, to be honest; we've just been buying frozen foods and frozen pizzas and nuggets and stuff because that's the most affordable and the easiest option at the moment’.

[Female, Turkish, Caring for mother]

Another carer, and something that came up for many, was limiting heating and water to keep costs down, which is particularly devastating for those with long‐term health conditions and older adults:

‘And even with the bills, you're limited to how many times you can have a shower in a week, how many times you can put heating on even though you're cold. We try to wrap up rather than put on thingy’.

[Female, British Asian, Caring for both parents and husband]

Another carer discussed how their financial situation impacted their ability to access childcare and social opportunities for their children through clubs showing how socioeconomic deprivation shaped the broader household experience:

‘I'm not frivolous in any way. I don't do holidays; don't do the coffees; don't do lunches; don't do clubs. So my kids don't do clubs because I can't afford it’.

[Female, White British, Caring for father]

Although access and availability varied among living locations of the participants, many praised the voluntary and community sector organisations, as they provided the practical support and helped relieve some of the financial pressure for them and the person they cared for:

‘The only organisations that help, or have helped me up to date, have been charities…it's taken a lot of pressure off me, like last week she gave me vouchers for Aldi. And I had no money at the end of last month, I had no money at all to pay for anything, and these vouchers turned up’.

[Female, White British, Caring for mother]

### 3.6 Social and Structural Barriers

#### 3.6.1 Lack of Social Mobility Opportunities

Hopes of social mobility being removed was mentioned by many, which was sometimes attributed to their financial situation and being unable to afford further education, but several times it was attributed to their caring role:

‘because obviously when you spend five, six years of doing a degree and all the time you have a future, you think “I'm going to have a good job, I'm going to going to be in a good house,” you know, have your own house and in a good area and stuff, but then you don't have any of that. Not because of any fault of your own, but that's just the way it works out sometimes, isn't it?’

[Female, Asian, Caring for in‐laws]

LTCs often develop at a younger age in people experiencing socioeconomic deprivation [25] and therefore the participant below became a carer in her teenage years, which meant balancing caring with their education:

‘Being in full‐time education and just managing my college work is stressful, on its own. So then I have to think about helping my mum, I also have to, pretty much, look after my little brother because my mum can't manage to do it’.

[Female, Turkish, Caring for mother)

A few carers mentioned working unsociable hours to ensure someone was always in the home or being employed on precarious employment contracts that offered no security:

‘But also, I think a lot of carers go onto zero‐hour contract work just because in our heads we think it's flexible but actually it's not good for us because it's very precarious, it causes more stress and you don't have pensions, like I didn't have a pension until I became part‐time’.

[Female, British Asian, Caring for both parents]

Those who had cultural barriers to content with as well, such as being a translator for the person they are caring for, meant they were unable to be in any paid employment as they had to accompany the person they cared for everywhere. In the case of the participant below, who was caring for his parents who did not speak English, he gave up work to attend these medical appointments and translate:

‘It does impact slightly because the time that I have to take out to take them to – increasingly to appointments now… I had to leave [place of employment]…if I envisioned ever doing paid work then I think it would impact at the moment, that as well. Even on a part‐time level I would struggle to balance both’.

[Male, Asian, Caring for both parents]

### 3.7 Area‐Based Barriers

Living in areas of deprivation impacted many of the carers’ lives, their ability to provide good quality care, and their access to services. One carer highlighted how they were unaware of any services that could support them or the person they cared for in their local area:

‘Maybe things like that are not advertised in our local surgery. I think that's one of the problems living in [Town], I think that access to information is not there. I don't know why but it's not there, I don't know of the services that we could access’.

[Female, Asian, Caring for in‐law parents]

Another participant reflected on their perceptions that living in a deprived area might have meant there would be better access to certain services for support, but soon found out this was not the case:

‘I was laughing because I was just thinking “Well, they've obviously got really good social care here,” because it's really poor; not a lot of money running around. But I can't get that. So I think there's a lot of disparities around; a lot of it's like what town you're in, what county you're in, you know’.

[Female, White British, Caring for adult daughter]

Several carers mentioned crime in their area that had impacted their role as a carer by avoiding going out to local parks and green spaces; this concern also came from the person they were caring for. One carer highlighted a more extreme example that meant the person they were caring for relocated due to being a victim of crime:

‘He lost the flat he had in [location] because these people came and kicked the main front door of the block in and they tried to get into his door, lucky they didn't get in the cops were called’.

[Female, Black British, Caring for ex‐spouse]

The carers in London in general discussed more situations where they used public transport compared to those living in Sheffield who discussed more use of taxis or not being able to access services due to travel restrictions:

‘And one of the best things ever is the free travel for over sixties in London, it's just superb, that must save me about £60 a week’.

[Female, White British, Caring for father]

### 3.8 Unheard by Health Care Professionals

Nearly all participants described delays to accessing necessary care or finding the correct diagnosis for those they cared for, often linked to a feeling of not being heard. These interactions acted as a barrier to carers understanding the care required and complexities of the medical conditions of those they cared for. One participant describes how not being listened to felt invalidating, which impacted their well‐being:

‘You know, we all want to be heard. People may not understand but just be heard is so ‐‐ because it's you know, it's testament you exist and what you're saying is valid. But when you're shut down or you're ignored, that just adds so much anxiety’.

[Female, Irish, Caring for father]

A few participants felt that some of the interactions they or the person they cared for had with health care professionals had been patronising, which led to a breakdown in the relationship between the carer and the health care professional. Many participants would then highlight the intelligence or the capacity of the person they cared for:

‘[Health care professionals are] very patronising when they talk to him and they go, “Oh, do you understand?” For god's sakes, this guy's got the sharpest brain. He's got a very Glaswegian “Aye”, do you know what I mean?’

[Female, White British, Caring for father]

Some carers from ethnic minority groups reported that within the NHS, in an attempt to be culturally competent, certain assumptions were made and as a result led to worse outcomes:

‘They make an assumption that everyone is from a Muslim background so they just plonk a halal meal in front of them. And I don't think they do that piece of work as to thinking, “Shall I ask the patient what they would like?”…So yeah, there is that. That culturally is a little bit of an issue’

[Female, British Asian, Caring for both parents]

One participant reflected on how remote appointments increased their confidence to ask questions, whilst also highlighting a ‘top‐down’ feeling with some health care professionals when in‐person:

‘You know when you're face‐to‐face with certain health professionals, there is that sort of top‐down sort of feeling. Whereas on Zoom it's a little bit hard to maintain that isn't it?’

[Female, British Asian, Caring for both parents]

### 3.9 The Emotional Challenges and Rewards

#### 3.9.1 Emotional Work

A prominent challenge for carers was managing the mental well‐being of the person they cared for whilst trying to look after their own emotional needs. People experiencing socioeconomic deprivation have greater prevalence of lower mental well‐being and higher mental illness [26] meaning carers from this population are more likely to have to manage more complex situations. Carers highlighting that providing support for mental health conditions and poorer well‐being require greater emotional work:

‘That self‐harm is a big thing, she's cut all over her body from her neck right down to her feet, not her head, and I find that really, really distressing’.

[Female, Irish, Caring for adult daughter]

Carers described the difficulty of acting as an emotional support to family members as challenging. Some explained that practical responsibilities are often easier to deal with:

‘One thing I found very, very, very hard in his care, it's his emotional needs… Because if it's practical, I can go through it; I've done it, I've done the job. But his emotional needs are very draining’

[Female, White British, Caring for father]

Many carers reported employing coping strategies or a desire for such mechanisms as a form of self‐management. Carers engaged in physical activities, support groups, and creative outlets to mitigate the emotional impact of caring. However, financial constraints often acted as barriers to access and uptake, affecting both their well‐being and the care they could provide:

‘Swimming and painting… they're the two things that just it's like deeply therapeutic for me, but I can't afford the swimming…I haven't been eating properly because I'm just trying to save money so that I can take my kid and myself for a swim’

[Female, White British, Caring for mother]

### 3.10 Meaningful Role

Whilst most of what carers discussed was around the challenges, all carers mentioned the positive sides of caring too. Caring for their family members was a meaningful experience, which gave a sense of purpose at times and many valued these positives:

‘There are plus sides to it, and the plus sides are that ‐‐ I sound like I'm defending my family here, but the plus side is when you're caring for other people the plus side is your heart gets bigger, it's as simple as that. And you're given the opportunity to put somebody else before yourself’.

[Female, White British, Caring for adult son]

Others found local groups to share their experiences or support their own mental health. Many had reflected their caring role led them to become member of these groups.

‘I belong to this group I discovered during lockdown, called Outsiders Music Sight and Sound, and everyone has a mental health challenge. And it's working with a music therapist, who is also a music producer, and that has been like a lifeline. So I've actually written songs about my experience, I'm a survivor, who cares for the carer?’

[Female, White British, Caring for adult daughter]

A few carers tried to use their difficult situations to try to make a difference to others. Experiences were discussed such as writing to their local MP, joining political groups or campaigning:

‘I do campaign a lot about adult social care because care workers…there is no formal qualification. If you were to look after an older adult or a vulnerable adult…there aren't many sort of hoops that you have to jump through to become one of those care workers’.

**Source:** [Female, British Asian, Caring for both parents]

Lastly, there were a few carers who reflected that because of their culture, caring is considered an honourable thing to do and they took comfort in that perception:

‘Because our culture and our religion as well, it makes us take on that responsibility and it reminds us of how noble that is’.

[Female, Asian, Caring for in‐law parents]

## 4 Discussion

### 4.1 Summary

The study explored the implications of socioeconomic deprivation on carer experiences and the ability to provide care and support. Three prominent themes were developed that focused on economic insecurity due in part to challenges with carer financial welfare, insecure housing and a ‘cost of living’ crisis in the United Kingdom. This insecurity led carers to sacrifice necessities such as healthy food, water and heating. The second theme describes the additional social and structural barriers this population face beyond financial difficulties such as a lack of social mobility opportunities, living in a deprived area with little resources and feeling unheard by professionals. The third theme pulled together the emotional challenges and rewards that come with caring such as the emotional work it takes to care for others who are often experiencing poor mental well‐being, but also how the carers find meaning within this role.

### 4.2 Comparison With Existing Literature

Carers experiencing socioeconomic deprivation faced additional challenges in their caring role such as managing insecure and unsuitable housing for themselves and the person they were caring for. Frequently moving house is known to dissolve local social relationships [27] leaving carers particularly vulnerable as those relationships with the community often supports their caring role [28]. Carer's allowance in the United Kingdom was challenging to navigate and even when it was received, it was not enough to live on leading to cutting back of necessities. Due to their caring role, most carers were unable to secure employment or education opportunities to improve their socioeconomic situation, which appeared worse for people who had to translate for the person they were caring for and therefore had to attend all appointments and outings. Research has previously shown the policies in England do not support carers to ensure they are not at risk of poverty or social exclusion [29]. Our research highlights the impact these policies are having on carers with financial limitations and stressors permeating into nearly every aspect of carers’ experiences, which have only been exacerbated by the UK cost‐of‐living crisis.

Carers were living in areas of deprivation, meaning higher crime, less access to resources and a general lower standard of living. Evidence has long shown living in deprivation is particularly detrimental to the health and well‐being of people experiencing poverty potentially due to their reliance on local resources [30, 31]. When carers did access services, such as health services, they often felt unheard or ignored by health professionals. This finding is consistent with previous research that described how people experience socioeconomic deprivation felt their care was impacted by their perceived status leading to feelings of distrust [18, 32]. Carers and health care professionals must work in partnership and our research highlights this is a potential barrier for this population and therefore the person they are caring for.

The carers of this population are more likely to be managing complex health conditions alongside challenging living situations [25], with our participants caring for people with several long‐term health conditions. The emotional work involved in managing LTCs has been highlighted in recent research [33]; this emotional work appears to be greater for carers of people experiencing socioeconomic deprivation. However, participants found their caring role meaningful and when they were faced with difficult situations, they often leant into this. Meaningful work has shown to be fundamental to well‐being [34]; however, it is often lower in people experiencing socioeconomic deprivation who do not have the same employment opportunities as more affluent people [35]. Therefore, despite the many challenges caring comes with, it also provided a protective factor for this population's well‐being.

### 4.3 Strengths and Limitations

The inclusion of carers experiencing socioeconomic deprivation is a strength of this study, as it provides a voice for an underserved population. Carer literature has so far focused on financial constraints and the impact on employment [14], but not specifically explored the multi‐dimensional nature of socioeconomic deprivation. Whilst the impact of caring on people from ethnic minority backgrounds has been reported [36, 37], the diversity of ethnicity in this study allowed us to explore cultural barriers that intersect with deprivation. However, our sample only included two males (out of 12) and one young adult (i.e., below the age of 24) meaning their experiences have not been explored in depth in this study.

### 4.4 Implications for Research and/or Practice

From a national policy level, the carer's allowance welfare currently offered in the United Kingdom needs reviewing urgently in terms of access and the amount paid as it does not support carers experiencing socioeconomic deprivation. At the local policy level, making areas of deprivation safer and ensuring there are sustainable resources available for carers and for people with long‐term health conditions would be hugely beneficial to the well‐being of this population. Carers experiencing socioeconomic deprivation need to be identified and highlighted in their medical records, so that appropriate support can be offered such as access to social prescribing [38]. Training for health care professionals on supporting people experiencing socioeconomic deprivation and the role of carers in self‐management of health conditions would be valuable to ensure carers feel heard and supported. Further research is needed to tailor appropriate resources and support for carers’ emotional well‐being, as well as support for caring for people with complex long‐term health conditions. It is clear carers experiencing socioeconomic deprivation have multiple pressures impacting on their ability to care. Supporting such carers is potentially cost‐effective given the care provided reduces demand for resources provided by the state.

## 5 Conclusion

Carers experience many challenges; however, those experiencing socioeconomic deprivation face additional and exacerbated difficulties. Our paper has highlighted the added emotional work and anxiety involved in caring for people experiencing socioeconomic deprivation, difficulties accessing welfare support and the sacrifices to necessities of living made due to their financial situations. Thorough development and evaluation of services and subsequent policy changes are essential to support this population and to reduce inequalities.

## Author Contributions

**Megan Armstrong:** conceptualisation, investigation, funding acquisition, writing – original draft, writing – review and editing, supervision, formal analysis, methodology, data curation. **Alma Jeri‐Wahrhaftig:** project administration, writing – original draft, writing – review and editing, investigation, methodology, formal analysis. **Abi Woodward:** writing – review and editing, supervision, formal analysis, methodology, data curation. **Danielle Nimmons, Carolyn A. Chew‐Graham, Joanne Protheroe, Fiona Stevenson and Nathan Davies:** conceptualisation, investigation, funding acquisition, writing – review and editing, formal analysis. **Kate Walters:** conceptualisation, investigation, funding acquisition, writing – review and editing, supervision, formal analysis.

## Ethics Statement

The Health Research Authority (HRA) (22/LO/0227) granted ethical approval.

## Conflicts of Interest

The authors declare no conflicts of interest.

## Supporting information

Supplementary material 1.

## Data Availability

The data that support the findings of this study are available from the corresponding author upon reasonable request.
