# Supplementary material for: Experiencing Socioeconomic Deprivation as a Carer in the United Kingdom: A Qualitative Study
Source: Health Expect. 2025 Nov 19;28(6):e70502. doi: 10.1111/hex.70502 (PMC12630546; doi:10.1111/hex.70502)
Supplement: Supplementary file 1 — Supplementary material 1. [file HEX-28-e70502-s001.docx]

**Carer Topic Guide: socioeconomic deprivation**

1. **Thinking about your caring role, can you talk me through what a typical week looks like for you?**

Do you have to work to a routine? (e.g., care plan, managing visits/appointments around your own needs - shopping, work, cleaning, childcare…)

1. **Due to the health conditions of the person you care for, can you tell me about anything specific in your caring role that you find challenging or difficult?**
2. **In what ways, if any, does your financial situation impact on your caring role and life in general?**

Able to undertake paid work/progress in career/education?

Carers allowance e.g. awareness of entitlement, able to claim benefits/manage renewals?

If applied for carers allowance but was turned down, why?

Cutting back in any areas (e.g., activities)

1. **Have you attended GP or other healthcare appointments with the person you care for? If so, can you give me and example of a time when you have had a good or not so good experience?**
2. **Can you tell me about any difficulties you have had (or are having) with accessing support or health services, either for yourself or the person you care for?**
3. **What additional support (if any) in the local area, are you and the person you care for receiving that helps with managing their health and wellbeing?**
4. **Can you tell me about anything that you currently do to look after your own health and wellbeing?**
